# RETRACTION: Integrated Bioinformatics-Based Identification of Potential Diagnostic Biomarkers Associated with Diabetic Foot Ulcer Development

**DOI:** 10.1155/jdr/9839207

**Published:** 2025-11-25

RETRACTION: L. Qian, Z. Xia, M. Zhang, et al., “Integrated Bioinformatics-Based Identification of Potential Diagnostic Biomarkers Associated with Diabetic Foot Ulcer Development,” *Journal of Diabetes Research*, 2021, 5445349, https://doi.org/10.1155/2021/5445349

The above article, published online on 01 September 2021 in Wiley Online Library (http://wileyonlinelibrary.com), has been retracted by agreement between the journal Associate Editor, Bright Starling Emerald, and John Wiley & Sons Ltd. The retraction has been agreed due to data concerns initially raised by an external reader. Following an investigation into these concerns, the Associate Editor has determined that the conclusions in the article are unreliable and it is therefore retracted.

The authors agree to the retraction.

